# Enhanced detection of low concentration volatile organic compounds using advanced doped zinc oxide sensors

**DOI:** 10.1039/d3ra03143h

**Published:** 2023-10-17

**Authors:** Majdi Benamara, Ahmadou Ly, Sonia Soltani, Manel Essid, Hassen Dahman, Ramzi Dhahri, Lassaad El Mir, Marc Debliquy, Driss Lahem

**Affiliations:** a Laboratory of Physics of Materials and Nanomaterials Applied at Environment (LaPhyMNE), Faculty of Sciences in Gabes, Gabes University 6072 Gabes Tunisia majdibenamara1@gmail.com; b Laboratory for Building Energy Materials and Components, Swiss Federal Laboratories for Materials Science and Technology (Empa) Überlandstrasse 129 8600 Dübendorf Switzerland; c Service de Sciences des Matériaux, Université de Mons Rue de l’Epargne 56 7000 Mons Belgium; d Department of Physics, College of Science and Arts, Qassim University Dariyah 58251 Saudi Arabia; e Department of Chemistry, College of Science, King Khalid University Abha 61413 Saudi Arabia; f Department of Physics, Faculty of Sciences and Arts, Najran University P. O. Box 1988 Najran 11001 Saudi Arabia; g Materia Nova, Materials R&D Centre Parc Initialis, Avenue Nicolas Copernic 3 7000 Mons Belgium

## Abstract

Pure zinc oxide nanoparticles, as well as those doped with 3% calcium, aluminum, and gallium, were synthesized using a sol–gel method and then deposited onto an alumina substrate for sensing tests. The resulting nanoparticles were characterized using a variety of techniques, including X-ray diffraction (XRD), scanning electron microscopy (SEM) equipped with energy dispersive X-ray analysis (EDX), transmission electron microscopy (TEM), UV-VIS-NIR absorption spectroscopy, and photoluminescence (PL) measurements, to examine their structural, morphological, and optical properties. The prepared nanoparticles were found to have the hexagonal wurtzite structure of ZnO with a *P*63*mC* space group. The UV-Vis-IR spectra showed that the samples are highly absorbent in the UV range, while the PL spectra confirmed the presence of many defects in the ZnO structure, such as oxygen vacancies and zinc interstitials. The doped samples exhibited more defects than the pure sample. SEM images of the deposited film surface showed agglomerates with a spherical shape and confirmed the nanometer scale size of our prepared samples, as corroborated by the TEM images. The EDX spectra indicated the high purity of the ZnO deposited films, with a high presence of Zn and O and the presence of the doped elements (Ca, Al, and Ga) in each doped sample. Sensing tests were performed on ZnO, Ca_3%_-doped ZnO (C3ZO), Al_3%_-doped ZnO (A3ZO), and Ga_3%_-doped ZnO (G3ZO) sensors in the presence of volatile organic compounds (VOCs) gases such as ethanol, formaldehyde, methanol, and acetone at low concentrations. The sensors exhibited high responses to low ppm level concentrations of the VOCs gases. At a low operational temperature of 250 °C, the C3ZO sensor had the highest response to 5 ppm of ethanol, methanol, and formaldehyde gases compared to the pure and other doped sensors. Additionally, the A3ZO sensor exhibited the highest response to acetone gas. In conclusion, our findings suggest that the doping of zinc oxide can enhance the low concentration detection of VOCs gases, with the C3ZO and A3ZO sensors showing the highest response to specific gases.

## Abstract 2

Pure zinc oxide nanoparticles, as well as those doped with 3% calcium, aluminum, and gallium, were synthesized using a sol–gel method and then deposited onto an alumina substrate for sensing tests.

## Graphical abstract

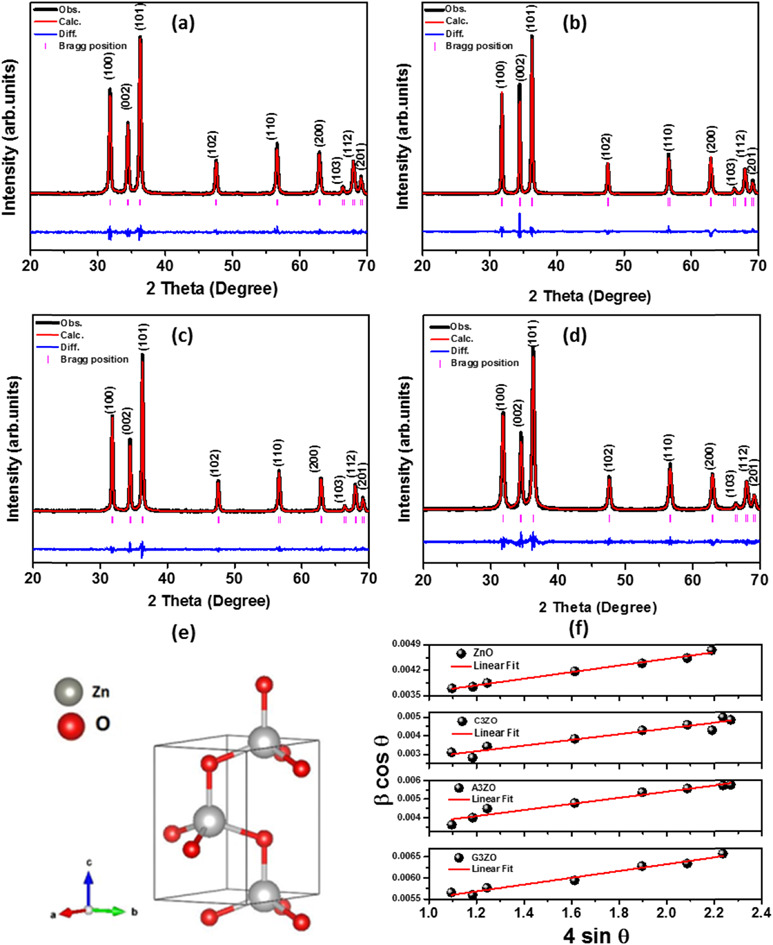

## 1. Introduction

Air pollution is a global problem in our world caused by the development of industry. Every year, three million people die due to environmental pollution, and according to recent statistics, this number could grow to more than six million by 2050 if the same trend in pollution emission levels continues.^1^ To see a solution to this problem, several researchers have been studying the construction of devices that allow the detection of toxic gases. Metal oxide-based gas sensors (MOX) are a technology that has attracted great attention in several fields, such as solar cells, electrical assemblies, gas sensors, *etc.* MOX have several important characteristics, such as being low cost, easy to use, and highly sensitive to various gases.^2,3^ The detection of volatile organic compounds (VOCs) is essential in industry and in the laboratory environment. VOCs in the human breath, such as acetone, ethanol, ammonia, ethane, and pentane, serve as biomarkers for several diseases.^4^ Typical levels of acetone in the breath of healthy humans range from 0.3 to 4 ppm,^5,6^ while this can increase to 40 ppm in adults that follow a ketogenic diet.^7^ In addition, there are other types of VOCs, such as methanol and formaldehyde, which can negatively affect human health. For example, exposure to a concentration of formaldehyde greater than 6 ppm can cause nasopharyngeal cancer, leukemia, and even lung cancer.^8^ Also, methanol is very toxic and often fatal to humans.^9^

Gas sensors based on semiconductor metal oxides (MOS) are the most promising because of their compact design, their low cost, their high sensitivity, and their applicability in the detection of VOCs in expired air. Most sensor materials, such as In_2_O_3_,^10^ SnO_2_,^11^ Fe_2_O_3_,^12,13^ ZnO,^14^ WO_3_,^15^ TiO_2_,^16^ Co_3_O_4_,^17^ and so on, have excellent gas detection properties. In particular, there are several reports where chemoresistive sensors, based on metal oxides, have been used for the detection of VOCs. Zinc oxide, which has several interesting properties, is a type II–VI semiconductor material with a high exciton binding energy of ∼60 meV and a wide band gap of 3.37 eV. As such, zinc oxide has received considerable attention due to its many applications in optoelectronics, gas sensors, solar cells, and electronics.^18,19^ Also, compared to other metal oxides, ZnO is quite attractive for its high thermal and chemical stability and its relatively simple preparation. The detection properties of sensors based on ZnO can be modulated by altering the particle morphology, crystal structure, and energy band structure. Li *et al.*^20^ report that a gas sensor based on ZnO nanowalls has a high acetone vapor detection response with rapid recovery and response times. Jaballah *et al.*^21^ showed that Ca-doped ZnO nanopowder elaborated *via* a sol–gel method has good formaldehyde sensing properties with a high response rate (5 ppm, 250 °C) and a low detection limit at each ppm concentration they examined. Zahmouli *et al.*^22^ proved that a sensor based on γ-Fe_2_O_3_/Al–ZnO nanocomposites, prepared using a sol–gel method, had a high sensitivity to acetone gas.

In this work, we present a study of the sensing properties of pure and doped ZnO materials in response to low concentrations of VOCs gases. The prepared samples were made using a modified sol–gel process. The zinc oxide was doped with aluminum, calcium, and gallium to enhance its sensing performance. The VOCs gases tested were acetone, methanol, ethanol, and formaldehyde. Structural, morphological, and optical proprieties were investigated to explain the sensing mechanism.

## 2. Experimental details

### 2.1. Synthesis of ZnO nanoparticles and characterization

A sol–gel method was used to synthesize the ZnO nanoparticles. We dissolved 16 g of zinc acetate dihydrate [Zn(CH_3_COO)_2_, 2H_2_O, 99%] in 112 mL of methanol. The solution was maintained under magnetic stirring until the precursor was dissolved. Then, we introduced the solutions into an autoclave with 220 mL of ethyl alcohol to dry the solution under the supercritical conditions of ethanol (*T*_c_ = 243 °C, *P*_c_ = 63.6 bar). The resulting powder was calcined in a muffle furnace under air at 400 °C for 2 hours.

Structural and morphological characterizations of the prepared samples were carried out using a Philips PW1710 X-ray diffractometer with CuKα1 radiation (1.54186 Å) and using field-emission scanning electron microscopy (FE-SEM, S4800II, Hitachi, Japan) with energy dispersive X-ray analysis (EDX). Transmission electron microscopy (TEM) was performed using a JEOL JEM 2010 electron microscope (LaB6 electron gun) equipped with a Gatan 794 Multi-Scan CCD camera and operating at 200 kV. A NanoLog Horiba modular spectrofluorometer was used to conduct the photoluminescence (PL) measurements to understand the optical properties and to investigate the defects found at the bandgap of the nanomaterial. The excitation light source used was a Xe lamp. The range of emission was between 350 and 750 nm at an excitation wavelength of 325 nm. Also, the UV-vis absorbance spectra were acquired using a UV-vis spectrophotometer (Shimadzu UV-3101PC).

### 2.2. Sensor fabrication and sensing tests

250 mg of the prepared nanopowder was dissolved in 2.25 mL of distilled water. Then, the mixture was sonicated by ultrasound until a uniform and dispersed solution was obtained. Subsequently, we deposited the homogeneous solution on alumina substrates (Al_2_O_3_) (C-MAC Micro Technology Company, Belgium) equipped with a pair of interdigitated gold electrodes and Pt heating elements, by a spray method. To stabilize the deposited film on the substrate, the sensor was heated for 1 hour at 400 °C.

Detection tests were performed in a homemade gas detection measurement system. We begin by injecting commercial synthetic air (79% N_2_ + 21% O_2_) into a Teflon chamber, which contains the sensor being tested, until the baseline is stable. After that, we inject the target gas at an appropriate concentration. The relative humidity (RH) and temperature were continuously monitored. The gas sensor response is defined as *S* = *R*_a_/*R*_g_ where *R*_a_ and *R*_g_ are the electrical resistance of the sensor in air and the target gas, respectively. Response/recovery times were evaluated at the 90% resistance change point after exposure to the target gas and air.

## 3. Results and discussions

### 3.1. Structural properties

**Fig. 1:**
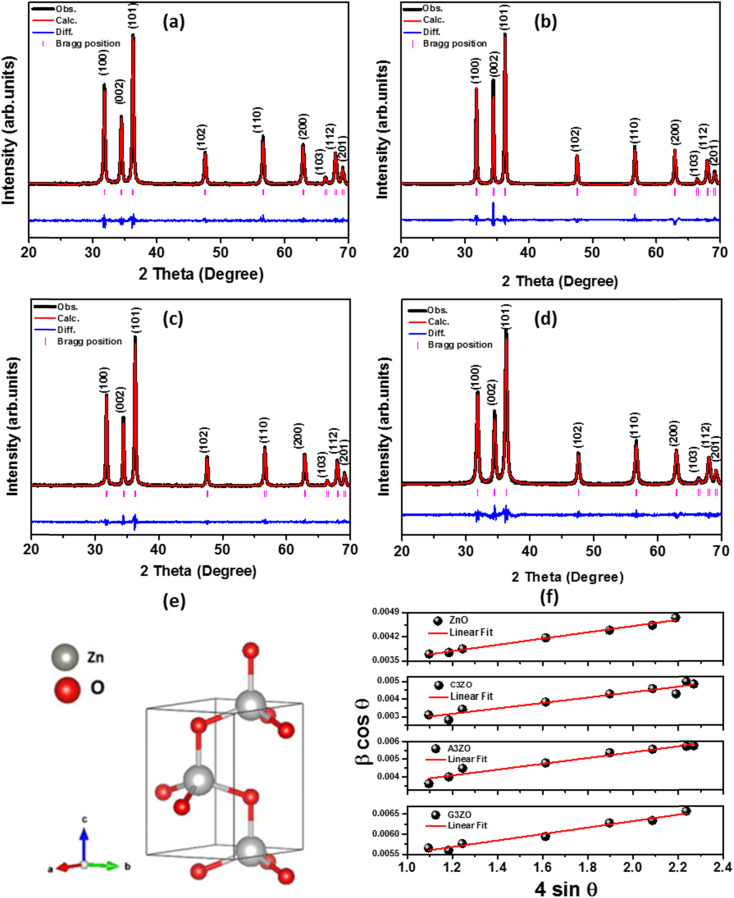
Rietveld refined X-ray diffraction patterns of the (a) ZnO, (b) C3ZO, (c) A3ZO, and (d) G3ZO nanoparticles. (e) ZnO unit cell and (f) the Williamson–Hall plots of the undoped and doped ZnO samples.

The crystallographic structures of the prepared samples of ZnO, Ca_3%_-doped ZnO (C3ZO), Al_3%_-doped ZnO (A3ZO), and Ga_3%_-doped ZnO (G3ZO) were obtained *via* X-ray diffraction analysis at room temperature, as presented in Fig. 1-a–d, respectively. All spectra were refined by the Rietveld method using the FullProf software. This method is based on the pseudo-Voigt function. The refinement indicates that all samples crystallize in the hexagonal structure with the *P*63*mC* space group. This indicates that doping with Ca, Al, and Ga does not induce a structural change in the ZnO structure. In addition, no distinct phase is observed in our samples. All fitting parameters are collected in Table 1 and the excellent goodness of fit, *χ*^2^, values indicate the good quality of the refinement. There is good agreement between the observed and the calculated plots. Fig. 1-f represents the Williamson–Hall plots. The micro-deformation (*ε*) and the average size of the crystallites (*D*) were estimated according to the Williamson–Hall model:^23^1
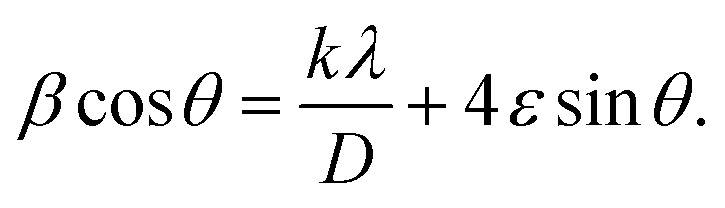


**Table 1:** Structural parameters of pure and doped ZnO

Compounds	ZnO	C3ZO	A3ZO	G3ZO
Crystal system	Hexagonal	Hexagonal	Hexagonal	Hexagonal
Space group	*P*63*mC*	*P*63*mC*	*P*63*mC*	*P*63*mC*
*a* (Å)	3.251	3.250	3.251	3.250
*b* (Å)	3.251	3.250	3.251	3.250
*c* (Å)	5.208	5.207	5.208	5.204
Cell volume *V* (Å^3^)	47.654	47.634	47.659	47.636
*R*_p_ (%)	6.360	6.206	7.160	5.739
*R*_wp_ (%)	8.490	6.732	9.780	7.133
*χ* ^2^	1.478	1.279	1.498	1.387
*D*_W–H_ (nm)	52	126	37	30
*ε*	9.1 10^−4^	17.8 10^−4^	16.5 10^−4^	8.7 10^−4^
*δ* (line nm^−2^)	81.1 10^−5^	65.4 10^−5^	116.2 10^−5^	13.3 10^−5^
*D*_SEM_ (nm)	38	68	23	35

The plots of (*β* cos *θ*) *versus* (4 sin *θ*) were fitted according to eqn (1). The *D* value is estimated from the slope of the extrapolation of the linear fit and the *ε* value is given by the fit slope. The obtained *D* values are equal to 52 nm, 126 nm, 37 nm, and 30 nm for the ZnO, C3ZO, A3ZO, and G3ZO samples, respectively. The obtained results show that doping with calcium increases the grain size more than doping with the other elements due to the difference in size of the radii of Ca^2+^ (*r* = 99 pm) and Zn^2+^ (*r* = 74 pm). Meanwhile, the smaller radii of Ga^2+^ (*r* = 62 pm) and Al^3+^ (*r* = 53 pm) cause a reduction of the crystallite size. In addition, the dislocation density (*δ*) was calculated using the following relationship:^24^2
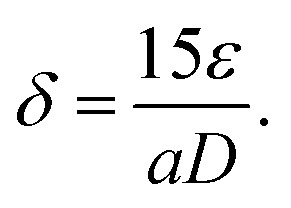


The calculated values of *D*, *ε*, and *δ* of the prepared samples are presented in Table 1. The high observed values of *δ*, present essentially in the doped samples, suggests that there are defects present in the structure of the prepared samples. These defects can improve the gas detection performance of the samples.

### 3.2. Morphological proprieties

**Fig. 2:**
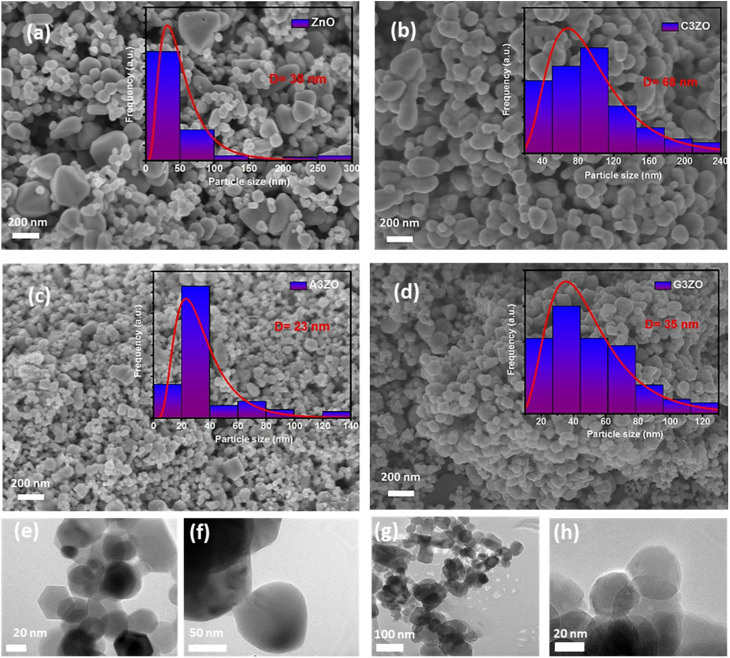
SEM images of (a) ZnO, (b) C3ZO, (c) A3ZO, and (d) G3ZO with diagrams of the particle distributions (inset). TEM images of the (e) ZnO, (f) C3ZO, (g) A3ZO, and (h) G3ZO nanoparticles.

The typical SEM micrographs of our samples ZnO, C3ZO, A3ZO, and G3ZO are displayed in Fig. 2-a–d, respectively, and show the surface morphology and the distribution of the grain size (*D*). The obtained pictures show the presence of agglomerates with a spherical form with an uneven distribution. The insets of Fig. 2 illustrate the Gaussian-fitted histograms calculated using the ImageJ software. The histograms indicate the distribution of average grain size in our samples. The average grain sizes determined by SEM are equal to 38, 68, 23, and 35 nm for ZnO, C3ZO, A3ZO, and G3ZO, respectively. All grain size values are collected in Table 1. The particle size values estimated from SEM are larger than those estimated from the W–H formula. Each grain is almost monocrystalline in nature. The SEM images show a smaller grain size for A3ZO, and G3ZO and larger grain sizes for C3ZO, thus confirming the observations obtained from the W–H method. Fig. 2-e–h present TEM images of the particle shapes of the ZnO, C3ZO, A3ZO, and G3ZO samples, respectively. The grain sizes, as evidenced by the TEM of the prepared samples, agree well with the SEM images and are less than 100 nm, thus confirming the nanometer scale of the prepared powders. The shapes of almost all the particles are spherical with the presence of some hexagonal nanoparticles also noted.

**Fig. 3:**
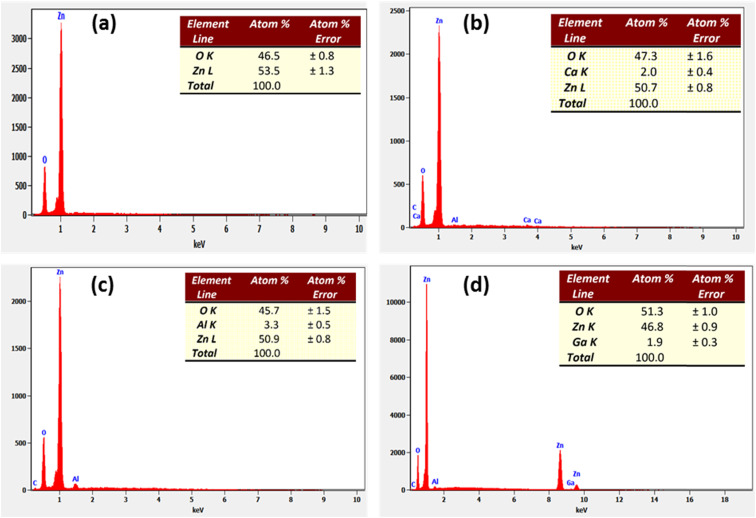
EDX spectra of the (a) ZnO, (b) C3ZO, (c) A3ZO, and (d) G3ZO nanoparticles.

Fig. 3-a–d show the EDX spectra of the ZnO, C3ZO, A3ZO, and G3ZO samples, respectively. This method is used to determine the purity and to confirm the presence of the constitutional elements in our samples. The EDX spectra prove the presence of all elements, such as Zn, O, Ca, Ga, and Al, in our compounds. It is worth noting that no constitutional elements were lost in our samples compositions. In addition, we note the presence of carbon and aluminum in the spectra. The carbon comes from the precursor compound and the aluminum from the sensor substrate.

### 3.3. Photoluminescence and UV-visible light absorption characterization

**Fig. 4:**
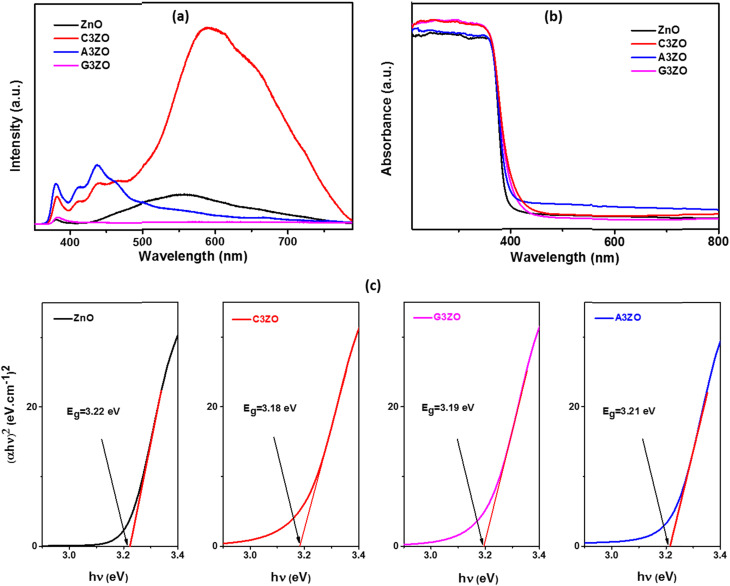
(a) PL spectra, (b) UV-visible-NIR absorption spectra, and (c) Tauc plots for ZnO, C3ZO, A3ZO, and G3ZO.

The PL spectra of the doped and pure ZnO samples were captured and are shown in Fig. 4-a. Pure zinc oxide has a large peak in the visible light region resulting from a deep-level emission (DLE), as well as a peak in the UV region that is focused on a near band edge (NBE) emission. The large visible band is linked to several intrinsic defects. Ca doping causes the NBE peak to move to shorter wavelengths, which may be related to the Moss–Burstein effect. The Fermi level and optical band gap are often increased in C3ZO nanostructures due to the replacement of the Zn^2+^ ions that are present in their network locations with Ca^2+^ ions. Ca doping induces a sharp rise in emission intensity. This is mostly due to the Ca^2+^ ions, which have a larger atomic radius (1.14 Å) than zinc ions, replacing Zn^2+^ ions, which have a smaller atomic radius (0.88 Å). The sites in the crystal structure are disrupted by this substitution, which also increases the number of defects present in the substance. This leads to an increase in the number of oxygen defects (V_o_), which has been linked to green emissions, and an increase in the number of interstitial zinc sites (Zn_i_), which has been linked to red emissions.^25,26^ Beyond that, there were visual differences between the PL emission spectra of the pure ZnO and Al-doped ZnO (A3ZO) samples. The UV emission band intensified while the green emission band steadily diminished in intensity. A new band with a center at 437 nm also developed, which we relate to a blue emission. This emission may result from intrinsic flaws or from the recombination of donor–acceptor pairs linked to the acceptor Al. Overall, our findings indicate that the strength of the blue band grew as the Al content increased, whereas the intensity of the green band decreased as the Al content increased.^27^ Ga-doped ZnO (G3ZO) exhibits a dramatic reduction in its green emission and an increase in the UV emission in its PL spectrum. Ga atoms occupy Zn vacancy defect positions and increase the donor-associated defect amounts in ZnO, resulting in intense UV emissions in the PL spectra.^28^

The absorbance spectra of ZnO, C3ZO, A3ZO, and G3ZO in the UV-visible-NIR range were examined and are displayed in Fig. 4-b. It was found that the absorbance decreased noticeably for wavelengths higher than 400 nm (in the visible and near infrared ranges) and that the absorbance was highest in the UV light range (400 nm). Such a redshift in the photosensitivity of the Ca-doped ZnO sample presumably resulted from the sub-band gap transitions produced by the incorporation of the Ca ions in the structure of ZnO, as evidenced by an absorption wavelength of the Ca-doped ZnO nanoparticles of 400–500 nm. According to the UV-vis absorption spectrum of A3ZO, the edge values of the AZO nanoparticles were blue-shifted in comparison to the ZnO nanoparticles, and the absorbance in the visible region improved primarily as a result of the deep levels in the ZnO bandgap caused by an increased concentration of defects.^2^ Ga doping makes it possible to increase the optical absorption rate in the visible range, thus revealing that the light absorption capacity is increased by the intercalation of the gallium atoms, resulting in easier transport of the charge carriers between the valence and conduction bands.

In order to determine the band gap energy of the prepared samples, the variation of (*αhυ*)^2^ as a function of the energy of the photons was studied (Fig. 4-c) according to Tauc's model:

3 *αhυ* = *A*(*hυ* − *E*_g_)^*n*^.

Here, *α*, *h*, *ν* and *A* are the absorption coefficient, the Planck constant, the photon frequency, and a constant, respectively. For materials with a direct band gap, the exponent “*n*” has a value of 1/2. The band gap energy (*E*_g_) for each sample can be calculated by extrapolating the linear portion of the curve to the *x*-axis. For ZnO, C3ZO, A3ZO, and G3ZO, the estimated *E*_g_ values are 3.22, 3.18, 3.21, and 3.19 eV, respectively. We see that the *E*_g_ of these samples falls off when a doping element is intercalated into the ZnO structure.

According to published research, a decrease in the energy of the band gap can be attributed to a drop in carrier concentration, an increase in tensile stress, and an increase in inter-band defects such oxygen vacancies.^29^

### 3.4. Gas sensing performance

**Fig. 5:**
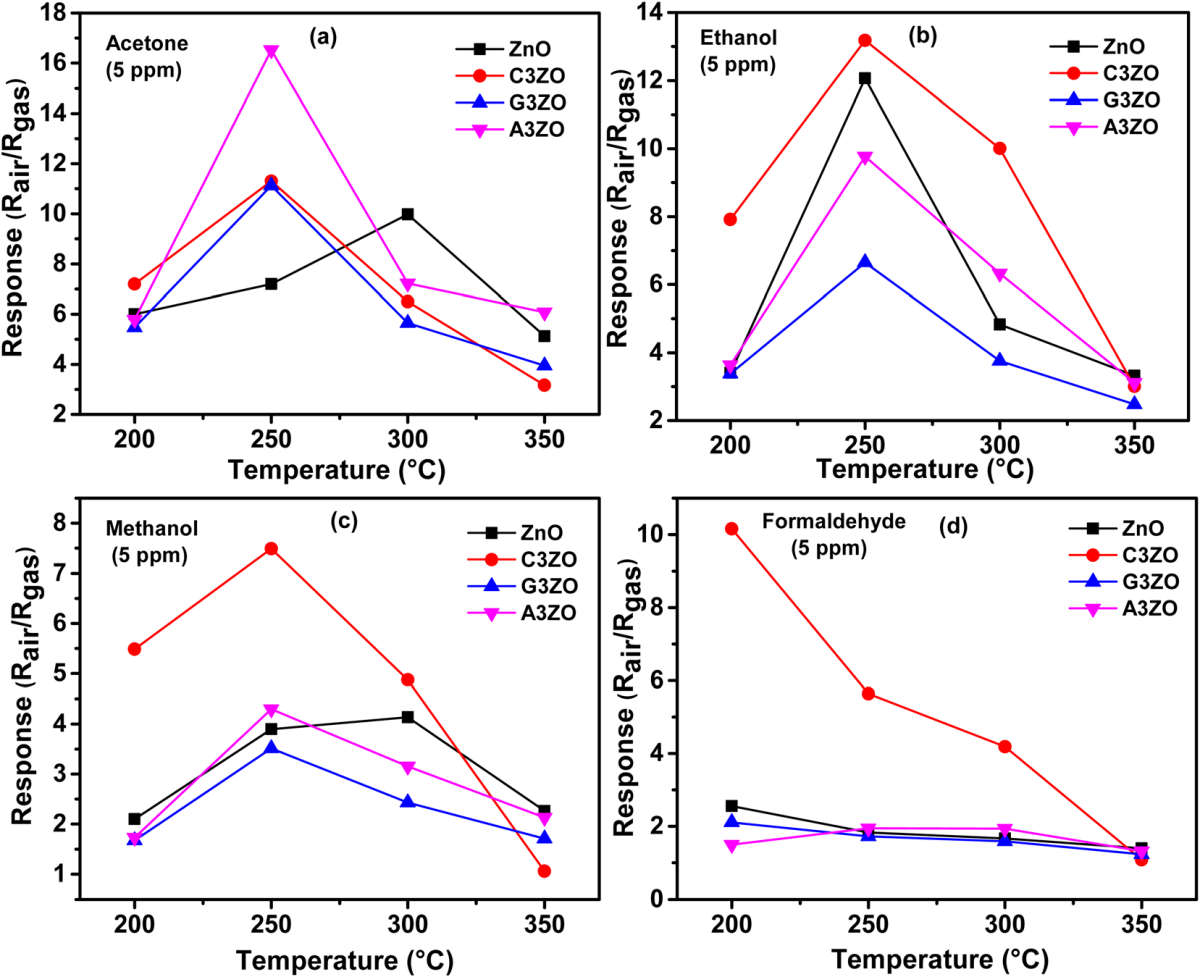
Sensor responses as a function of temperature in the presence of 5 ppm of (a) acetone, (b) ethanol, (c) methanol, and (d) formaldehyde gases for the ZnO, C3ZO, G3ZO, and A3ZO samples.

The prepared sensors were tested in the presence of 5 ppm of acetone, ethanol, methanol, and formaldehyde gases with 50% RH at different temperatures ranging from 200 to 350 °C. The sensor responses to VOCs gases, such as acetone, ethanol, methanol, and formaldehyde, as a function of temperature are presented in Fig. 5-a–d, respectively. The curves indicate that the highest responses are at an operational temperature of 250 °C.

**Fig. 6:**
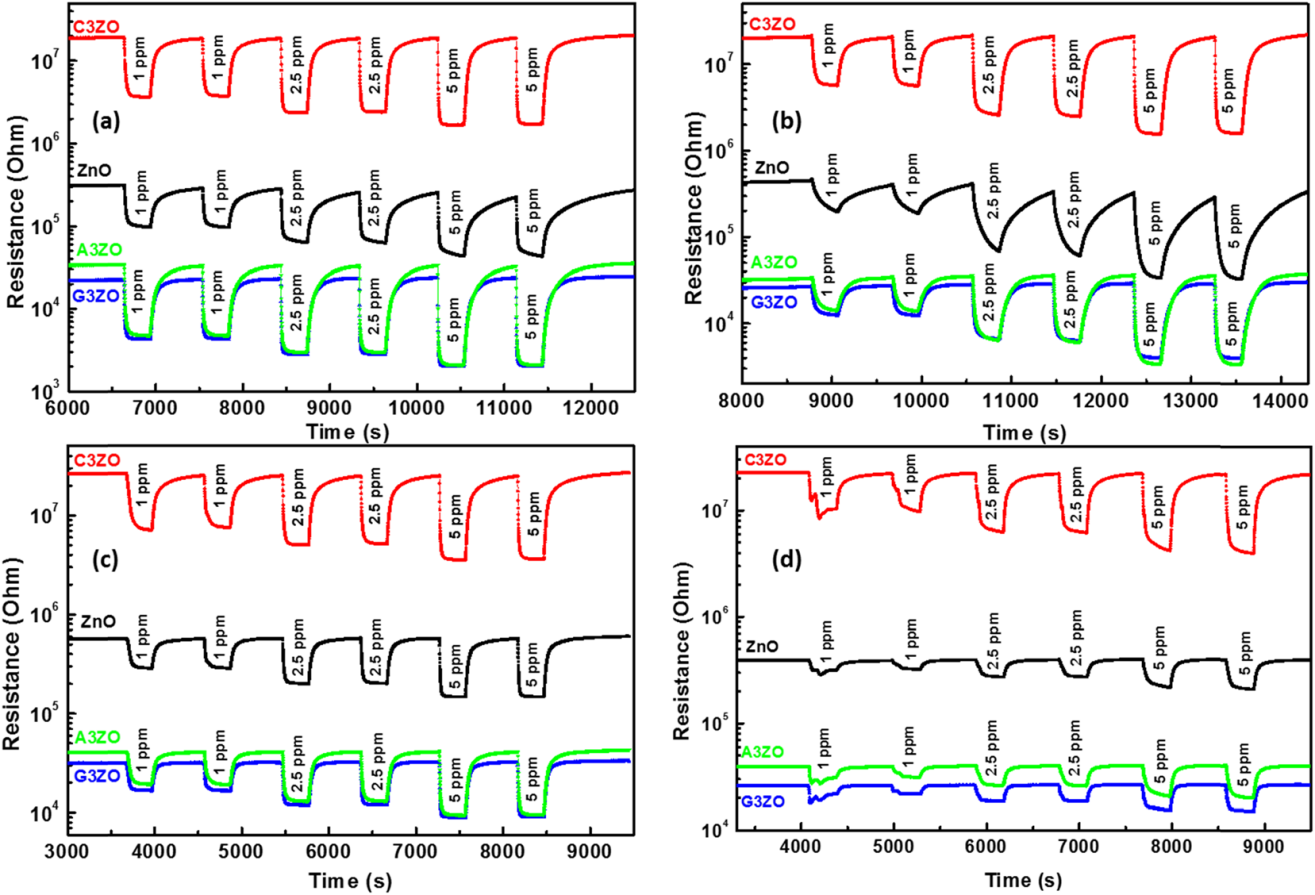
Electrical resistance *versus* time for the ZnO, C3ZO, A3ZO, and G3ZO sensors at 250 °C and under 50% RH in response to (a) acetone, (b) ethanol, (c) methanol, and (d) formaldehyde gas at different concentrations.

The doped sensors present a response to VOCs gases better than the pure sample. The best response to acetone gas came from the A3ZO sensor and the best sensor response to ethanol, methanol, and formaldehyde gas came from the C3ZO sensor. In addition, we note that doping ZnO with Ga enhances the response to acetone gas. This improvement is related to doping in general because it creates defects in the internal structure of the ZnO. These defects are generally interstitial and oxygen vacancies, which have the most important role in the gas detection mechanism.^30^ The improved acetone gas responsiveness of the A3ZO sensor may be attributed to the following factors. First off, the A3ZO NPs have a smaller size than the ZnO NPs and a nanospherical structure, factors which both point to a larger specific area. Gas molecules will diffuse more readily as a result, and they will interact with the adsorbed oxygen on the A3ZO surface more positively. This implies that it may be possible to significantly increase the concentration of adsorbed species. Secondly, the presence of oxygen vacancies, whose adsorption energy is probably three times greater than that of the optimal position, could have a major impact on the sensor response.^31^ Unoccupied oxygen sites in the A3ZO structure are utilized as an adsorption site to improve gas detection. As a result, gas molecules can react with oxygen species that have been adsorbed at open oxygen sites. When there are several gas molecules adsorbed on the surface, there is a lot of electron mobility. Despite the electron flow remaining unaltered, this causes the percolation channel to widen and the electron-depleted layer to contract. The electrical resistances of the prepared sensors in the presence of ethanol, methanol, formaldehyde, and acetone gas at different gas concentrations at 250 °C and under a humidity of 50% are presented in Fig. 6-a–d, respectively. We investigated the sensor response to various low concentrations of each gas (1 ppm, 2.5 ppm, and 5 ppm) to see what effect low VOCs gas concentrations had on the sensor responses. Each concentration was injected two times consecutively to understand the reproducibility of our sensors. The responses to each concentration are almost consistent for all gases which indicates the high reproducibility of our sensors to low ppm concentrations of VOCs gases. Note that for the prepared sensors, upon exposure to the target gas, the resistance decreased rapidly to an equilibrium level and then it returned to baseline when humid air was reinjected into the Teflon chamber. This behavior is in good agreement with the behavior associated with the detection mechanism of n-type semiconductors. At an operating temperature of 250 °C, we conducted comparisons between all the sensors that were investigated. This highlighted that the Ca-doped ZnO nanoparticles have excellent potential for detecting ethanol, methanol, and formaldehyde fumes at low concentrations. It is evidently true that after each pulse, the signal of the Ca-doped films resets to its initial value. This behavior demonstrates the reversibility of the surface layer adsorption of the tested gases. The baseline resistances of the Ca-doped ZnO sample are higher than those of the undoped ZnO, it should be noted. The decrease in the carrier concentration of the Ca-doped sample is associated with this rise in resistance. As opposed to the other sensors based on doped samples, the baseline resistance of the Ca-doped ZnO sample increases following Ca doping by 3%, indicating a larger baseline resistance. The increased resistance may be primarily responsible for the higher responsiveness of the C3ZO sensor. The increased resistance is most likely due to the many flaws on the surface, which allow for the chemisorbtion and ionized adsorption of numerous oxygen molecules. The sensor response and baseline resistance both follow the same pattern. As more Ca is inserted, they grow. Particularly at low gas concentrations, C3ZO has ethanol, methanol, and formaldehyde detecting abilities that are competitive in comparison to other literature studies. High basicity is an attribute of calcium and on the surface of ZnO it produces centers with simple character. The high adsorption of the tested gases is facilitated by these sites, and therefore the good sensing performance was caused by the Ca doping. Therefore, C3ZO could be helpful for the creation of a high-efficiency ethanol, methanol, and formaldehyde gas sensor for real-world applications due to its straightforward synthesis process and outstanding gas detection capabilities.

**Fig. 7:**
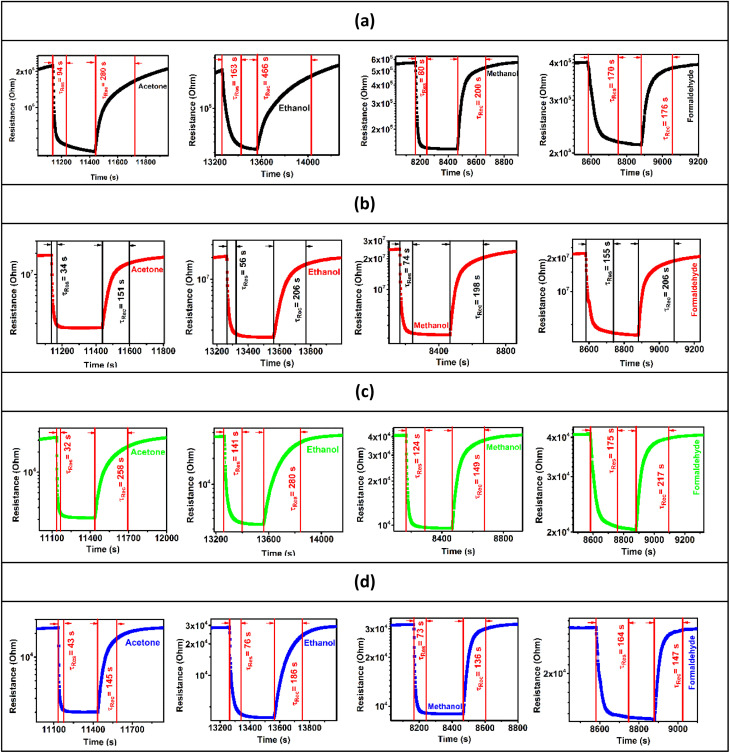
Base resistances as a function of time in response to acetone, ethanol, methanol, and formaldehyde gas for the (a) ZnO, (b) C3ZO, (c) A3ZO, and (d) G3ZO sensors.

The response/recovery times were estimated from the base resistance variation of the pure and doped ZnO samples in response to air and the target gases. Fig. 7-a–d present the resistance as a function of time in response to air and the target gases (acetone, ethanol, methanol, and formaldehyde) at a concentration of 5 ppm at 250 °C and at 50% RH, for ZnO, C3ZO, A3ZO, and G3ZO, respectively. By comparing the pure and doped samples, we can see that the doped samples have faster response and recovery times than pure ZnO. This behavior can enhance the efficacity of sensors. Thus, doping improves the most important parameters (responses and response/recovery times) required for gas detection.

**Fig. 8:**
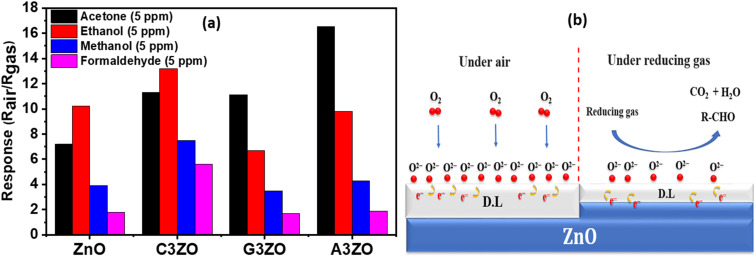
(a) Sensors responses towards 5 ppm of the target gases at 250 °C and at 50% RH. (b) Schematic of the gas sensing process under air and reducing gas atmospheres.

Selectivity is a parameter that describes the capacity of a sensor to distinguish between particular input signals. For sensors operating in complicated settings, selectivity is crucial.^32^ By observing the reaction of a transmitter to a specific gas concentration, we may calculate selectivity. This response is the same as the sensor response brought on by a certain target gas concentration. Cross-sensitivity lowers the measurement repeatability and reliability, making this attribute crucial for monitoring numerous gas applications. Good sensitivity and high selectivity are necessary for the perfect sensor. To increase selectivity, modified layer and gas sensor arrays are integrated. The selectivity diagrams of the pure and doped ZnO sensors are presented in Fig. 8-a and show the sensor responses towards 5 ppm of acetone, ethanol, methanol, and formaldehyde gas at 250 °C and at 50% RH. Pure ZnO has a higher selectivity for ethanol gas compared to the other tested gases. Also, we note that the C3ZO sensor presents a higher selectivity for ethanol than the other gases and that its selectivity to acetone gas is marginally smaller than its selectivity to ethanol.

The A3ZO sensor has a better sensitivity toward acetone vapor than to formaldehyde, ethanol, and methanol, showing superior acetone vapor selectivity. By including Al in the ZnO structure, the reaction to acetone is enhanced while the reactivity to formaldehyde, ethanol, and methanol is minimized. The extraordinary selectivity of the Al-doped ZnO nanoparticles may be attributed to the difference in the dipole moments of the target gases, which is significantly greater for acetone (2.91 D) than for ethanol (1.66 D), methanol (1.70 D), and formaldehyde (2.33 D). Other factors, such as the size and molecular weight of the gas molecules, also affect how the sensor responds.^33^

### 3.5. Gas sensing mechanism

Oxygen adsorption and desorption on the ZnO deposit layers have been demonstrated experimentally by exposure to humid air at a working temperature ranging from 200 °C to 350 °C. The interaction of atmospheric oxygen with the ZnO surface forms a layer of charged oxygen species which traps electrons from most of the material. The electrons captured during this process are removed from the conduction band and confined in the adsorbed oxygen species at the surface.^34^ Therefore, a space charge zone is formed because the presence of negative surface charges leads to web bending defined by a potential barrier to the surface. This restricts the weak electrons and subsequently allows a decrease in conductivity.

Fig. 6 shows a change in resistance during alternate exposure to moist air and organic vapor (ethanol, formaldehyde, methanol, and acetone) at different concentrations. These measurements were carried out at an optimal temperature of 300 °C. The evolution of the response as a function of the working temperature shows an optimum temperature for each gas. There is a direct correlation between oxygen adsorption and the detection of the reducing gas. In fact, the responses to the reducing gases exhibit a similar evolution and neighboring optimum temperatures. Increasing the operating temperature under the optimum temperature improves the oxygen adsorption and therefore the response to VOCs. In contrast, the desorption of all previously adsorbed ionic oxygen species occurs at high temperatures, which explains the decrease in the response to reducing gases above the optimum temperature.

For the identification of the ionosorption of oxygen species, we performed measurements on the ZnO sensor at an operating temperature of 250 °C by varying the concentrations of ethanol, formaldehyde, methanol, and acetone. Thus, the adsorbed oxygen ionization equations can be written by the following equations:

4 O_2(gas)_ → O_2(ads)_

5 O_2(ads)_ + 4e^−^ → 2O_(ads)_^2−^.

When we inject a reducing gas into the tested cell, the molecules of the gas will react with the oxygenated species on the surface of the material which causes a reinjection of electrons in our detection material and subsequently the potential barrier will be reduced and the conductivity will be increased. The reactions between the VOCs gases (ethanol, formaldehyde, methanol, and acetone) and the oxygen species are expressed by the following equations:^35^

6 C_2_H_5_OH_(gas)_ + O_(ads)_^2−^ → CH_3_CHO_(ads)_ + H_2_O + 2e^−^

7 HCOH_(gas)_ + 2O_(ads)_^2−^ → CO_2(gas)_ + H_2_O + 4e^−^

8 CH_3_OH_(gas)_ + O_(ads)_^2−^ → H_2_CO_(gas)_ + H_2_O + 2e^−^

9 CH_3_COCH_3(gas)_ + 8O_(ads)_^2−^ → 3CO_2(gas)_ + 3H_2_O + 16e^−^.

The general reaction between the oxygen species and the main element of the reducing gas is shown in more detail in the mechanism presented in Fig. 8-b.

### 3.6. Comparative study of prepared samples *vs.* other materials

To evaluate the performance of our prepared samples in comparison to other materials, we conducted a comprehensive analysis by referencing the literature on the responses of other materials to VOCs, specifically acetone, ethanol, methanol, and formaldehyde. We considered key parameters such as operational temperature and gas concentration, which are detailed in Table 2.

**Table 2:** Comparison of VOCs (acetone, ethanol, methanol, and formaldehyde) gas-sensing performance of sensors in this study with other materials

Material	Gas concentration	Operational temperature	Response	Reference
**Acetone gas**				
ZnO spheres	1 ppm	230 °C	1.5	36
CuO/ZnO	2.5 ppm	220 °C	3.5	37
ZnO	5 ppm	300 °C	2.3	38
rGO/CuO–ZnO	10 ppm	340 °C	9.4	39
ZnFe_2_O_4_/ZnO microstructures	50 ppm	320 °C	10	40
Al-doped ZnO	5 ppm	250 °C	16.5	This work

**Ethanol gas**				
Mg-doped ZnO	5 ppm	300 °C	4.0	41
Ag-doped ZnO	1 ppm	300 °C	0.9	42
EuFeO_3_ nanoparticles	500 ppm	295 °C	5.0	43
CuO nanoleaves	100 ppm	260 °C	3.0	44
SnO_2_–CuO	100 ppm	320 °C	8.0	45
Ca-doped ZnO	5 ppm	250 °C	13.2	This work

**Methanol gas**				
SnO_2_	100 ppm	270 °C	3.8	46
α-Fe_2_O_3_	50 ppm	340 °C	2.5	47
Al-doped ZnO thin layer	500 ppm	275 °C	1.44	48
CuO thin layer	550 ppm	350 °C	0.12	49
SnO_2_ nanosheets	100 ppm	275 °C	3.0	50
Ca-doped ZnO	5 ppm	250 °C	7.5	This work

**Formaldehyde gas**				
SnO_2_ nanowires	10 ppm	270 °C	2.5	51
ZnSnO_3_	50 ppm	270 °C	5.0	52
Ag–LaFeO_3_	5 ppm	230 °C	4.8	53
Ag–TiO_2_	200 ppm	360 °C	3.7	54
SnO_2_/Fe_2_O_3_	20 ppm	220 °C	4.5	55
Ca-doped ZnO	5 ppm	250 °C	5.6	This work

Our most effective sensor for detecting acetone gas was found to be ZnO doped with 3% Al, exhibiting superior sensing characteristics when compared to other sensors based on ZnO. Furthermore, for ethanol, methanol, and formaldehyde sensors with low operational temperatures and exceptional sensitivity, ZnO doped with 3% Ca emerged as the top performer. A comparison with existing literature reveals that our prepared sensors outperformed others in terms of sensing characteristics, particularly for ethanol gas detection, surpassing Mg and Ag-doped ZnO, SnO_2_–CuO, and CuO-based sensors. Additionally, it demonstrated superior capabilities in detecting methanol and formaldehyde when compared to sensors based on SnO_2_, CuO, LaFeO_3_, TiO_2_, and Fe_2_O_3_.

## 4. Conclusions

Pure and doped zinc oxide powders were prepared *via* a sol–gel route and were characterized using X-ray diffraction (XRD), scanning electron microscopy (SEM) equipped with energy dispersive X-ray analysis (EDX), transmission electron microscopy (TEM), UV-VIS-NIR absorption spectroscopy, and photoluminescence (PL) measurements. The prepared samples had the hexagonal wurtzite structure of the ZnO material. We have sprayed the synthesized powders on an alumina substrate. The SEM images of the deposited films showed agglomerates with a spherical shape which confirmed the nanometer scale size of our samples whilst the EDX spectra indicated the high purity of the ZnO deposited film. The EDX analysis also showed the presence of the doping elements Ca, Al, and Ga in the structures of the Ca_3%_-doped ZnO (C3ZO), Al_3%_-doped ZnO (A3ZO), and Ga_3%_-doped ZnO (G3ZO) samples. The UV-Vis-IR spectra indicate a high absorption in the UV range and the estimation of the band gap energies indicates values that range from 3.18 to 3.22 eV where the highest value relates to the pure sample and the lowest value relates to C3ZO, indicating that doping can create a defect inter-band. The PL spectra indicated the presence of a peak in the UV region that is focused on a near band emission (NBE) (deep-level emission) and a large visible band linked to several intrinsic defects in our structure, such as Zn interstitial (Zn_i_) and oxygen vacancies (V_o_). Ca doping caused the NBE peak to move to shorter wavelengths which is related to the Moss–Burstein effect. A3ZO presents an intensified UV emission band while the green emission band steadily diminished in intensity. G3ZO exhibits a reduction in the green emission and an increase in UV emission. We have investigated the VOCs (ethanol, formaldehyde, methanol, and acetone) sensing capabilities of the elaborated sensors at low concentrations. The sensors exhibit a high response to low ppm concentrations of VOCs gases at a low operational temperature of 250 °C and at a humidity of 50%. Doping ZnO with Ca enhances the responses to ethanol, methanol, and formaldehyde gases and doping ZnO with Al ameliorates the sensitivity to acetone gas with an increased selectivity to this gas.

## Conflicts of interest

The authors declare that they have no known competing financial interests or personal relationships that could have appeared to influence the work reported in this paper.

## Supplementary Material
